# Beam Wander Restrained by Nonlinearity of Femtosecond Laser Filament in Air

**DOI:** 10.3390/s22134995

**Published:** 2022-07-02

**Authors:** Jiewei Guo, Lu Sun, Jinpei Liu, Binpeng Shang, Shishi Tao, Nan Zhang, Lie Lin, Zhi Zhang

**Affiliations:** 1 Institute of Modern Optics, Nankai University, Tianjin 300350, China; 1120200106@mail.nankai.edu.cn (J.G.); lusun@nankai.edu.cn (L.S.); liujinpei@mail.nankai.edu.cn (J.L.); 1120200107@mail.nankai.edu.cn (B.S.); 2120200253@mail.nankai.edu.cn (S.T.); zhangn@nankai.edu.cn (N.Z.); linlie@nankai.edu.cn (L.L.); 2 Tianjin Key Laboratory of Micro-Scale Optical Information Science and Technology, Tianjin 300350, China; 3 Tianjin Key Laboratory of Optoelectronic Sensor and Sensing Network Technology, Tianjin 300350, China

**Keywords:** femtosecond laser filamentation, turbulence, beam wander, filament LIDAR

## Abstract

The filamentation process under atmospheric turbulence is critical to its remote-sensing application. The effects of turbulence intensity and location on the spatial distribution of femtosecond laser filaments in the air were studied. The experimental results show that the nonlinear effect of the filament can restrain the beam wander. When the turbulence intensity was $3.31\times 10^{-13}\;{\mathrm{cm}}^{-2/3}$, the mean deviation of the wander of the filament center was only 27% of that of the linear transmitted beam. The change in turbulence location would lead to a change in the standard deviation of the beam centroid drift. Results also show that the filament length would be shortened, and that the filament would end up earlier in a turbulent environment. Since the filamentation-based LIDAR has been highly expected as an evolution multitrace pollutant remote-sensing technique, the study promotes our understanding of how turbulence influences filamentation and advances atmospheric remote sensing by applying a filament.

## 1. Introduction

Filamentation induced by the nonlinear propagation of femtosecond laser pulses in the air has attracted wide interest in the field of atmospheric science, taking into account its potential application in remote sensing [1,2]. If the beam reaches a critical power (e.g., $P_{cr}=3.77\lambda^{2}/8\pi n_{0}n_{1}$ for Gaussian beams) [3], femtosecond laser pulses can propagate long distances without significant loss of peak intensity in the process of filamentation because a long stable plasma column (tens of meters to several kilometers) occurs at the self-focal region with an ultrabroad spectrum bandwidth [4] and a clamped intensity of approximately 5 × 10^13^ W/cm^2^ [5]. Due to the high clamped intensity of the filament, most molecules can undergo multiphoton tunnel ionization and fragmentation that further result in the emissions of characteristic fingerprint fluorescence [6,7]. Therefore, by using LIDAR technology [8,9], remote filament-induced breakdown spectroscopy can effectively detect the complex constituents of molecules and atoms present in the ambient atmosphere [10,11]. An important factor to be taken into account during remote sensing is the interaction of the propagated laser pulse with a perturbed atmosphere both before and during filamentation.

Turbulence causes fluctuations in air density that lead to fluctuations in the refractive index of air and distort the optical waves. Distortions lead to the significant blurring, scintillation, and wander of the laser beam. Wave-front distortions are also expected to perturb the dynamic balance between Kerr self-focusing and plasma defocusing in the filaments. Experimental and theoretical studies were devoted to the influence of air turbulence on filament distance [12,13], filament survival rate [14,15], transverse filament wandering [16,17], filament spectral characteristics [18], optical pulse broadening [19], the enhancement of multifilament generation and filament-induced fluorescence [20,21], triggering filamentation using turbulence [22], and beam shaping that suppresses turbulence [23]. Trivial changes in filamentation have a great impact on sensitivity during remote sensing. The studies mentioned above showed that filaments become shorter and have transverse drift, and single filaments become multifilaments under turbulent conditions. However, the influence of turbulence position change on beam drift and filament formation was not clear, which is very important for the filament in remote sensing of air pollution. At the same time, whether the regulation of turbulence on the beam is consistent in linear and nonlinear transmission is of great significance to solve these problems.

In this paper, the effects of turbulence location and turbulence intensity on the horizontal and vertical drift of beam centroids are studied under linear and nonlinear propagation conditions. The nonlinearity of femtosecond laser filament could suppress the modulation effect of turbulence on the beam. At the same time, the relationship between the beginning and ending points of femtosecond laser filaments and turbulent position is studied. The studies are helpful in improving the accuracy of remote air pollution detection based on LIDAR.

## 2. Experimental Setup and Methods

**Figure 1:**
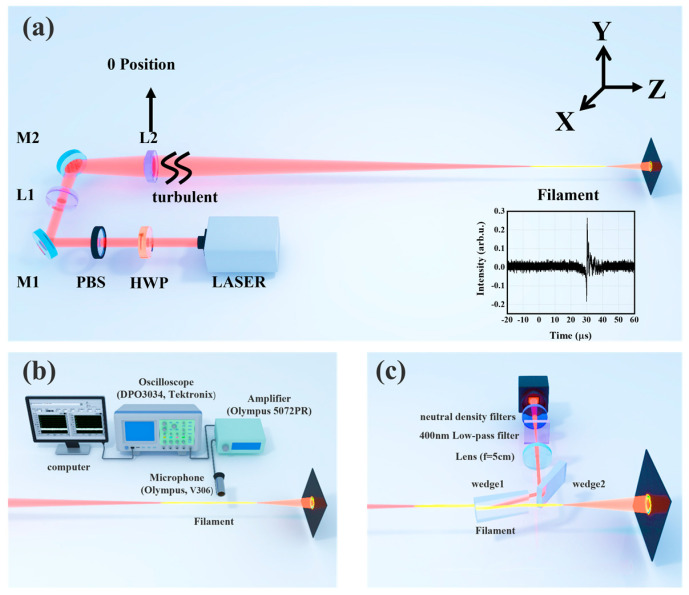
(**a**) Schematic diagram of the experimental device. Inset is a time-domain diagram of the filament-excited ultrasonic signal; (**b**) experimental apparatus for recording ultrasonic signal induced by filament under turbulent conditions; (**c**) experimental apparatus for recording the central drift of filaments under turbulent conditions.

The experimental setup is shown in Figure 1a. A Ti: sapphire femtosecond laser amplifier system (Legend, Coherent Inc., Santa Clara, CA, USA) was employed to generate 4.6 mJ, 500 Hz laser pulses with a central wavelength of 800 nm. High peak powers are typically accessed through a short pulse and thus a large bandwidth. The effects of group-velocity dispersion (GVD) in the atmosphere are critical to long-distance propagation, and a 50 fs transform-limited pulse would double in length within the first 100 m of propagation in air [24]. We chose a typical pulse duration for the filamentation experiment [17,25], which was 50 fs (full width at half maximum, FWHM), and the laser beam input diameter at the 1/e^2^ of maximal light intensity was 4 mm. An achromatic half-wave plate (HWP) and a polarization beam splitter (PBS) were used to control the energy. The polarization of the source was horizontal, and the light behind PBS was also horizontally polarized. By varying the distance between concave lens L1 (f = −10 cm) and planoconvex lens L2 (f = 50 cm), a filament was produced beginning from a distance of about 9.7 m concerning the planoconvex lens. At the same time, we defined the position of the planoconvex lens as the initial 0 positions, and artificial turbulence was introduced locally at the 0 positions by vertical airflow from a turbulent blower. The turbulent blower was a high-speed centrifugal turbo blower (1.2 kW) that changes the radial kinetic energy of the impeller to axial kinetic energy. The width of the turbulence zone was approximately 35 cm along the propagation. During the experiment, we changed the turbulent position with a step width of 35 cm along the propagation direction.

The filament was measured with the setup shown in Figure 1b. During photoionization, free electrons were ejected with high kinetic energy in the order of eV, which corresponded to an initial electron temperature of the order of 10^4^–10^5^ K [26]. The energy transfer between the hot free electron gas and the heavy species in the ambient gas occurred by collisions resulting in a hot gas column after the recombination of the plasma. The expansion of such a hot gas column led to an acoustic wave (AW) emission that could be detected by microphone. In our experiment, an ultrasonic probe (V306, Olympus. Ltd., Shenzhen, Chian) placed at a distance of 1 cm perpendicular to the propagation axis was used to collect the ultrasonic signal generated by the filament. An ultrasonic pulse receiver (5072PR, Olympus.td., Shenzhen, China) was used to amplify the ultrasonic signal detected by the microphone, which was then displayed on a digital phosphor oscilloscope (DPO3034, Tektronix Inc., Shanghai, China), as shown in the inset in Figure 1. The ultrasonic detection system (composed of a computer, oscilloscope, ultrasonic pulse receiver, microphone) was fixed onto a motorized precision translation stage that moved along the steel rail in parallel with the filament. The oscilloscope selected the acquisition mode and recorded the peak value of the acoustic signal at different positions of the optical filament through LABVIEW programming.

We built a simple imaging system to record the central beam position under different turbulent conditions, as shown in Figure 1c. Two fused silica wedges were inserted into the laser beam path, both at grazing angles, yielding a reflectivity of about 10% at each front surface. Therefore, after two surface reflections, the laser intensity was reduced to approximately 1%. The cross-sections of the laser beam were then detected by a CCD camera through a calibrated 1:4 image setup. The exposure time of the CCD was set as 1 ms to capture a single pulse for each picture. Various neutral density filters were placed in front of the CCD camera to further attenuate the laser intensity.

The cross-sectional spot of the femtosecond laser filament would wander in space due to turbulence, and the center of the spot would shift from the central optical axis. Due to the randomness of turbulence, the resulting beam wander was also random. We collected many data regarding the wander position of the spot center under the action of turbulence, and then analyzed the statistical law of variation in the filament wandering with the turbulence conditions. The beam wander was quantified by calculating the standard deviation of the beam center position along the horizontal and vertical directions. The beam center coordinates ($x_{c}$, $y_{c}$) were calculated according to:

(1) $$x_{c}=\frac{\sum_{x}\sum_{y}x\cdot S\left(x,y\right)}{\sum_{x}\sum_{y}S\left(x,y\right)}\;\;\;y_{c}=\frac{\sum_{x}\sum_{y}y\cdot S\left(x,y\right)}{\sum_{x}\sum_{y}S\left(x,y\right)}$$

where *S* indicates the intensity of the pixel.

**Figure 2:**
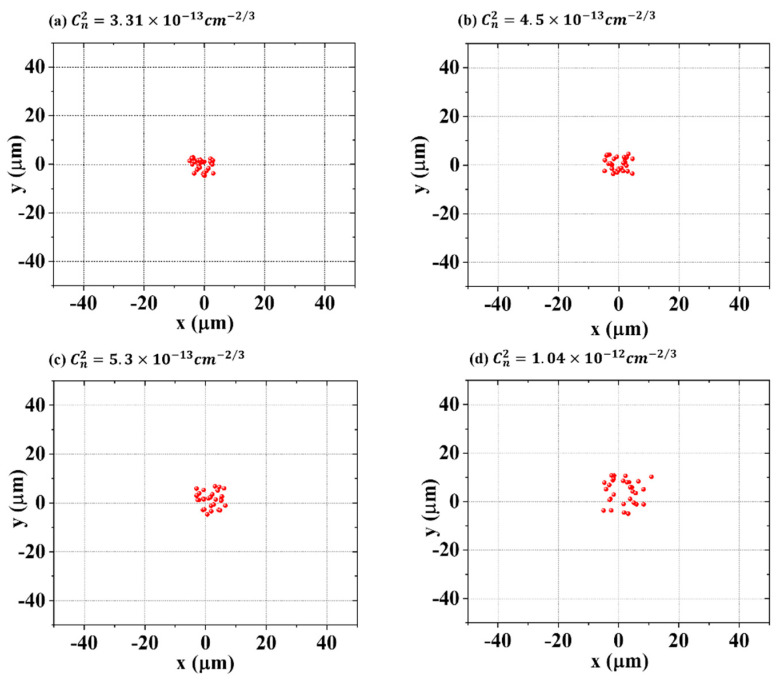
Spatial displacement of beam in turbulent air with various structural constants.

In our experiment, the strength of the local turbulence applied to the beam was maintained constant, i.e., the distance between the turbulent blower and the laser beam, and the parameters did not change during the measurements. The local turbulence was characterized by refractive index structure constant $C_{n}^{2}$, defined as $C_{n}^{2}=\frac{\langle {\left[n\left(r\right)-n\left(r+\Delta r\right)\right]}^{2}\rangle }{\Delta r^{\frac{2}{3}}}$ in Kolmogorov turbulence theory. $C_{n}^{2}$ can be determined experimentally by measuring the pointing stability of the He–Ne laser [27] using the expression $C_{n}^{2}=\sigma^{2}\phi^{1/3}/2.91L$, where $\sigma^{2}$ was the standard deviation of the angle of arrival. The beam diameter and the length of the turbulence region are denoted by ϕ and L, respectively. The air flow to generate turbulence was in the vertical direction, so the vertical $C_{n}^{2}$ and the horizontal $C_{n}^{2}$ may be different. However, as we can see from the Figure 2, the dispersion degree of the spot centroid in horizontal and vertical directions was close, so we could use the same $C_{n}^{2}$ for the horizontal and vertical comparisons. We quantified the intensity of the artificial turbulence, and the refractive index structure constants were $3.31\times 10^{-13}\;{\mathrm{cm}}^{-2/3}$, $4.5\times 10^{-13}{\mathrm{cm}}^{-2/3}$, $5.3\times 10^{-13}\;{\mathrm{cm}}^{-2/3}$, $1.04\times 10^{-12}\;{\mathrm{cm}}^{-2/3}$, which relatively correspond to atmospheric turbulence [28].

## 3. Results and Discussion

**Figure 3:**
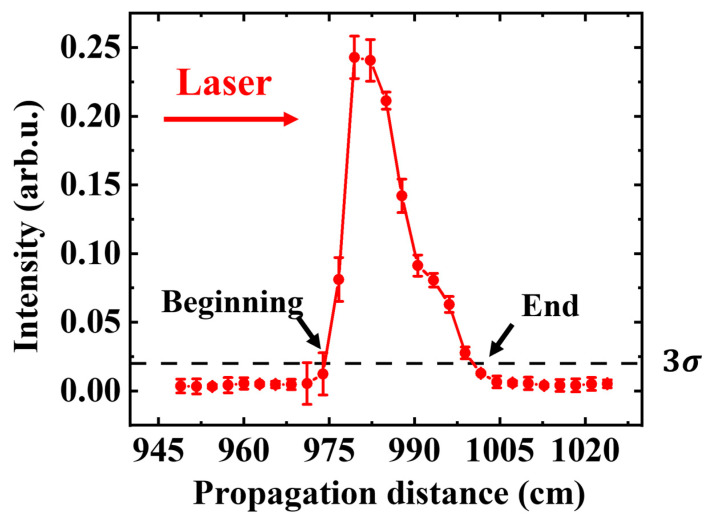
Peak amplitude of the acoustic signal at different positions in the absence of artificial turbulence (the red line represents the 3 times standard deviations of ambient noise (3σ)).

Figure 3 shows the acoustic intensity curve of ultrasonic signal intensity varying with transmission distance in the absence of artificial turbulence. First, an acoustic signal was detected 950 cm from the center of the L2 lens. Then, the microphone detected the intensity of the sound signal with a step length of 2.5 cm through the uniform motion of the electric slide rail, keeping the distance between the microphone and the wire unchanged, and stopping the detection when it was 1025 cm away from the center of the L2 lens. To reduce the influence of the microphone position on the directional error, the measurement in Figure 3 is the average result of five measurements. This variation in peak amplitude can be tracked to accurately determine the spatial extent of the filaments. Several groups of ambient noise were measured, 3 times standard deviation was calculated, and a 3 sigma standard line was plotted. The beginning and ending positions of the sound intensity line were determined by the intersection of the 3σ standard line and the sound intensity curve. The filament started at 974.2 cm and ended at 999.2 cm.

**Figure 4:**
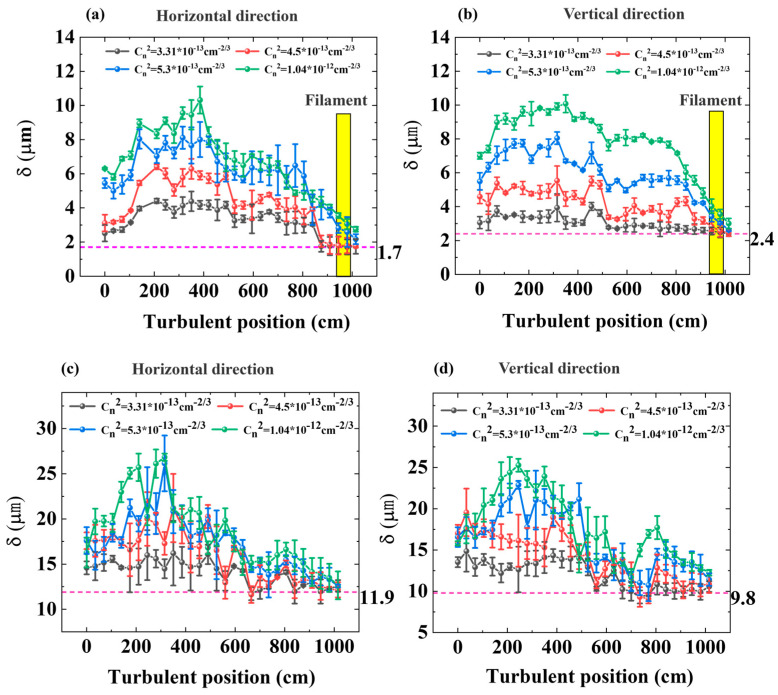
(**a**,**b**) Horizontal and vertical standard deviations of the beam center vary with the turbulence position under four turbulence strengths. Nonlinear transmission yellow rectangles represent the filamentation areas); (**c**,**d**) linear transmission. The pink baseline (dashed line) represents the horizontal and vertical standard deviations of the beam center under linear and nonlinear propagation conditions without artificial turbulence.

Turbulence at different positions had a complicated influence on the dynamics of long-distance filamentation. The perpendicular flow of a turbulent blower was used to generate strong local turbulence, and the effect on the spatial evolution of the filamentation was investigated. In total, 200 images were recorded with a CCD camera at each location and each turbulence strength condition. Then, the standard deviation of the beam position was calculated with Formula (1). As shown in Figure 4a,b below, the influence of different turbulence positions on the deviation of the filament center in the horizontal and vertical directions was studied, in which the yellow rectangles represent the filamentation areas. By rotating the half-wave plate in the experimental device, the femtosecond laser’s energy was reduced to 0.01 μW, which was not enough to form filaments in the air. Thereafter, the effects of different turbulent positions on the horizontal and vertical direction deviation of the beam center under linear transmission conditions were studied, as shown in Figure 4c,d. In the absence of artificial turbulence, the turbulence of the laboratory environment also leads to beam wander. The pink baseline (dashed line) represents the horizontal and vertical standard deviations of the beam center under linear and nonlinear propagation conditions without artificial turbulence, in which $C_{n}^{2}$ was $7.98\times 10^{-14}\;{\mathrm{cm}}^{-2/3}$. The standard deviation of beam center position δ was 11.9 and 9.8 μm in the horizontal and vertical directions for linear transmission, and 1.7 and 2.4 μm in the horizontal and vertical directions for nonlinear transmission.

Under the same turbulence, the horizontal and vertical standard deviations of the center of the filament first increased and then decreased, as the turbulence area was closer to the filament. Taking the turbulence intensity of $1.04\times 10^{-12}\;{\mathrm{cm}}^{-2/3}$ as an example, for nonlinear transmission, the standard deviation in the horizontal direction was 6.3 μm when the turbulence action position was at the specified position of 0 cm. Then, as the turbulence action region moved along the propagation direction, the standard deviation gradually increased to the largest value of 10.3 μm at 385 cm. Then, the standard deviation decreased gradually. The standard deviation was 2.75 μm when the turbulence position was 1015 cm, which was close to the value when artificial turbulence was not introduced. δ in the vertical direction changed with the turbulent position in a similar way as δ in the horizontal direction, as shown in Figure 4b. For linear transmission, the statistical results of the standard deviation of the wander of the beam showed the same rule.

Moreover, the horizontal and vertical standard deviations of the filament center increased with the augmented turbulence intensity. This trend was consistent with the results in [17]. Figure 4a shows that, as the turbulence intensity increased from $3.31\times 10^{-13}\;{\mathrm{cm}}^{-2/3}$ to $1.04\times 10^{-12}\;{\mathrm{cm}}^{-2/3}$ at the position of 385 cm, the standard deviation increased from 4.18 to 10.31 for nonlinear transmission. A similar tendency could also be found for linear transmission, but the deviation was much more significant. Figure 4c shows that, at the position of 385 cm, as the intensity of the turbulence increased, the standard deviation increased from 14.49 to 26.8 μm. Figure 4 shows that, when the turbulence was near the focal point of the system, the standard deviation of the beam wander did not change significantly with the increase in turbulence intensity, regardless of linear or nonlinear transmission.

**Figure 5:**
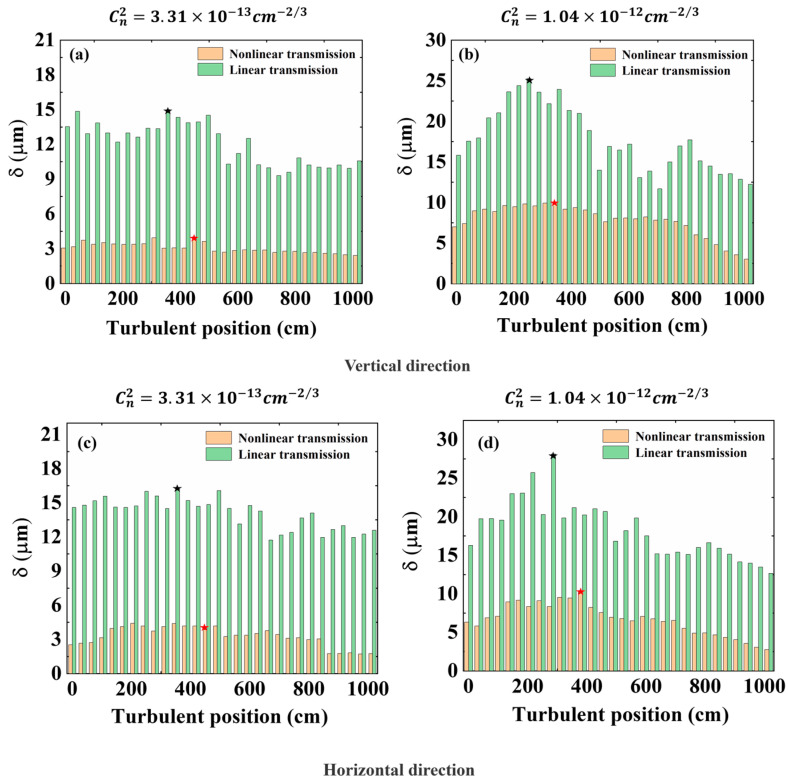
The standard deviation of beam wander in linear and nonlinear transmission at the same turbulent position. (**a**,**b**) Vertical direction; (**c**,**d**) horizontal direction; red pentacle, maximal value of δ in nonlinear transmission; black pentacle, maximal value of δ in nonlinear transmission.

In addition, the standard deviation of the beam wander obtained in linear and nonlinear transmission was different. Experimental results are shown in Figure 5, and we compared the beam wander standard deviations under the turbulence intensities of $3.31\times 10^{-13}\;{\mathrm{cm}}^{-2/3}$ and $1.04\times 10^{-12}\;{\mathrm{cm}}^{-2/3}$. Figure 5a,b show the wander standard deviation of the beam centroid in the horizontal direction, and Figure 5c,d show the wander standard deviation in the vertical direction. The green histogram shows the experimental results of linear transmission, and the orange histogram shows the experimental results of nonlinear transmission. Figure 5 shows that the green histogram representing nonlinear transmission was always higher than the orange histogram representing linear transmission in the process of turbulence location moving from 0 to 1000 cm. Taking the turbulence intensity of $3.31\times 10^{-13}\;{\mathrm{cm}}^{-2/3}$ as an example, Figure 5a shows that, in the process of turbulent position movement, the standard deviation of the filament’s center drift at the focal point was 4.0 μm in the case of nonlinear transmission. In the case of linear transmission, the maximal standard deviation of the beam centroid drift at the focus was 14.8 μm. The standard deviation of the filaments centroid drift in nonlinear transmission was 27.02% of the standard deviation of the beam centroid drift in linear transmission. When the turbulence intensity was $1.04\times 10^{-12}\;{\mathrm{cm}}^{-2/3}$, Figure 5b shows that the standard deviation of the filaments centroid drift in nonlinear transmission was 39.6% of the standard deviation of the beam centroid drift in linear transmission. In the vertical direction, as shown in Figure 5c,d, the above scores were 27.1% and 36.6%. Compared with the linear transmission, turbulence had a lesser effect on the deviation of thee beam center drift in nonlinear transmission. Previous work showed that the filament was very resistant to turbulence once it was formed [17]. Strong nonlinear effected occurring in the filament core, especially multiphoton absorption, were a driving force that overridden turbulence effects. Moreover, many of the basic phenomena that occur during the propagation of ultrashort laser pulses were connected with the fact that the refractive index has a nonlinear contribution that depends on the intensity of the pulse. Compared with the linear transmission, we mainly also need to analyze the optical Kerr effect in nonlinear transmission. In this case, the nonlinear refractive index change had the form $n_{nl}=△n_{kr}=n_{2}I$, where $n_{2}$ was the nonlinear refractive index, and *I* was the laser intensity. According to the expression of $n_{nl}=△n_{kr}=n_{2}I$, we could quantitatively and roughly estimate the refractive index value caused by the self-focusing effect to be $1\times 10^{-5}$. However, according to the expression of $\bar{{\left[n\left(x+r\right)-n\left(x\right)\right]}^{2}}=C_{n}^{2}r^{2/3}$ [20], we could quantitatively and roughly estimate the refractive index value caused by turbulence effect as $3.2\times 10^{-6}$. We could conclude that the self-focusing effect exceeded the turbulence effect. Due to the existence of the self-focusing effect, the beam wander caused by the turbulence effect could be inhibited.

**Figure 6:**
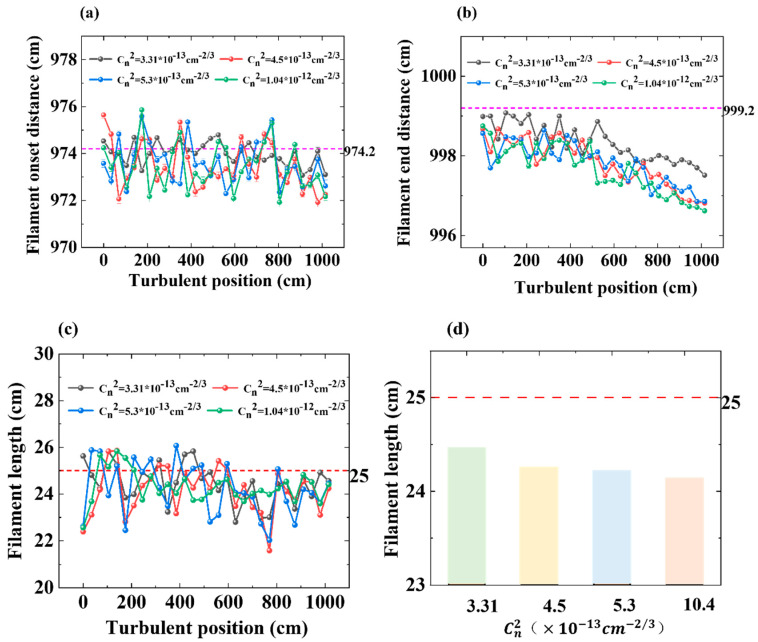
(**a**) The onset distance of the filament varied with the turbulence position under the four turbulence strengths; (**b**) the end distance of the filament varies with the turbulence position under the four turbulence strengths; (**c**) filament length varies with the turbulence position under the four turbulence strengths; (**d**) mean filament length varies with the turbulence position under the four turbulence strengths. The pink baseline (dotted line) shows the beginning and ending positions of the filaments in the absence of artificial turbulence and the red baseline (dotted) line shows the mean length of the filament without artificial turbulence.

Lastly, we studied the effect of the turbulence position at the beginning and ending positions of filaments, and the experimental results are shown in Figure 6a,b. The pink baseline (dotted line) in Figure 6 shows the beginning and ending positions of the filaments in the absence of artificial turbulence. As Figure 6d shows, the red baseline (dotted) line shows the mean length of the filament without artificial turbulence. We could find that, under the influence of turbulence, the filament was a little shorter. Because of the turbulence, the beginning position of the filament was moved a little forward. However, the ending position of the filament was even more sensitive to the turbulent position along the propagation direction. When the position of turbulence was closer to the filament, the end of the filament moved towards the lens. This result is consistent with previously reported simulation results [29]. With the increased intensity of the turbulence, the self-focusing of the filaments and the dynamic balance of the plasma trifocal were more easily broken, and the whole length of the filament was shortened. Due to the inhomogeneity of the refractive index of the optical medium caused by turbulence, the background energy pool was dispersed by turbulence in the early stage of filament formation, and the interference of the dispersed energy then formed a multifilament. Therefore, the shortening of thee filament length was mainly due to the loss of background energy.

## 4. Conclusions

In summary, we studied the spatial distribution of femtosecond laser filaments under the influence of turbulence. When turbulence had occurred before the filament was formed, it strongly affected the transverse wandering of the filament. If turbulence was applied to the filament, the filament was almost insensitive to turbulence. In addition, the length of the filament was shortened, and the end position of the filament was advanced under turbulent conditions. More importantly, we showed that the nonlinear phenomenon of femtosecond laser filamentation could suppress the wander of the beam perpendicular to the propagation plane due to turbulence. When the turbulence intensity was $3.31\times 10^{-13}\;{\mathrm{cm}}^{-2/3}$, the average deviation of the center drift was only 27% of that of the linearly transmitted beam. When the turbulence intensity was $1.04\times 10^{-12}\;{\mathrm{cm}}^{-2/3}$, the average deviation of the central drift of the filament was only 38.1% of that of the linearly transmitted beam. The result is valuable for the study of femtosecond LIDAR remote detection in complex atmospheric environments.

## Data Availability

The data presented in this study are available on request from the corresponding author.
